# Near-field optical microscopy of femtosecond-laser-reshaped silver nanoparticles in dielectric matrix

**DOI:** 10.1186/1556-276X-7-315

**Published:** 2012-06-19

**Authors:** Moritz Beleites, Christian Matyssek, Hans-Helmuth Blaschek, Gerhard Seifert

**Affiliations:** 1 Physics Institute, Martin Luther University of Halle-Wittenberg, D-06099, Germany; 2 Max Planck Institute of Microstructure Physics, , D-06120, Germany; 3 Fraunhofer Institute for Mechanics of Materials, IWM Halle, , D-06120, Walter-Hülse-Straße 1, Germany; 4 Centre for Innovation Competence SiLi-nano, Martin Luther University of Halle-Wittenberg, D-06099, Germany

**Keywords:** NSOM, Nanoparticles, Silver, Surface plasmon resonance, 68.37.Uv, 78.67.Bf, 61.46.Df.

## Abstract

Samples containing single silver nanoparticles have been irradiated by intense femtosecond laser pulses to gain a persistent transformation of their shape to ellipsoidal forms. Irradiated and non-irradiated regions of these samples have been analyzed by microscope spectrometry as well as near-field scanning optical microscopy (NSOM) with several wavelengths and different linear polarizations. The results show the outstanding capability of NSOM technique to detect the individual shape of transformed metallic nanoparticles and to analyze their orientation and aspect ratio.

## Background

Metallic nanoparticles (NPs) have been the subject of numerous optical studies for more than 20 years, mostly because of their very strong linear and nonlinear interaction with light in the spectral regions of their surface plasmon resonance (SPR). Especially for silver spheres in dielectric matrices, the SPR is located at the blue edge of the visible spectrum, well separated from the interband transitions in UV [1]. For elongated particles, the SPR with light polarized along the longer axis of the NP experiences a shift towards longer wavelengths; with increasing aspect ratio, this SPR may move throughout the visible into the NIR spectral range [2]. It has been demonstrated previously that low-concentrated Ag NPs embedded in glass can be transformed to prolate spheroidal shapes by irradiation with several hundred, linearly polarized femtosecond laser pulses of appropriately high energy density [3-5]. So far, however, the spectral analysis of the reshaped particles has been restricted to macroscopic studies [6,7] with implicit averaging over a large number of NPs with different sizes and shapes. In this letter, we demonstrate that for femtosecond-laser-deformed Ag NPs in an AlOx matrix, the individual shape of single NPs can be analyzed with the help of near-field scanning optical microscopy using different wavelengths and polarization directions.

## Methods

The samples used for this study have been prepared using commercially available spherical silver NPs in aqueous solution (BBInternational Silver Colloid). A mean particle diameter of 40 nm was selected because this promised the best comparability to previous works on Ag NPs embedded in glass [4-7]. The liquid containing the silver NPs was applied to thoroughly cleaned and dried substrates in drops of a few microliter, and the water was allowed to evaporate at room temperature. Finally, a 40-nm cover layer of aluminum oxide was prepared by atomic layer deposition (ALD). For comparison of near-field scanning optical microscopy (NSOM) transmission effects, similar samples were prepared using polystyrene (PS) NPs (Thermo Scientific ‘Nanosphere’ size standards, also 40 nm in diameter) instead of Ag NPs. Femtosecond irradiation was conducted using a frequency-doubled Yb:KGW laser system with 300 fs pulse length at a wavelength of 515 nm. We employed irradiation parameters comparable to a previous work on Ag-glass nanocomposites (approximately 500 pulses per spot, peak pulse intensity around 1 TW/cm^2^). Subsequent to the irradiation, the samples were annealed at 150°C for 60 min to remove possibly created defects in the matrix [6]. Conventional extinction spectra of selected laser-irradiated as well as non-irradiated reference regions were recorded using a microscope spectrometer. Each of these areas had a size of 30×30 *μ*m^2^. We performed near-field optical microscopy using an aperture-type NSOM (cantilever-probe-based WITec alpha300 S, WITec GmbH, Ulm, Germany) in transmission mode. Various laser sources with different wavelengths in the range of 405 to 785 nm were used, coupled to the NSOM via an optical fiber. Due to the uniform thickness of ALD-prepared aluminum oxide layer, the size of any individual Ag NP can be obtained from the simultaneously measured topography data (height difference between the highest spot on top of a particle and the surrounding flat surface).

**Figure 1:**
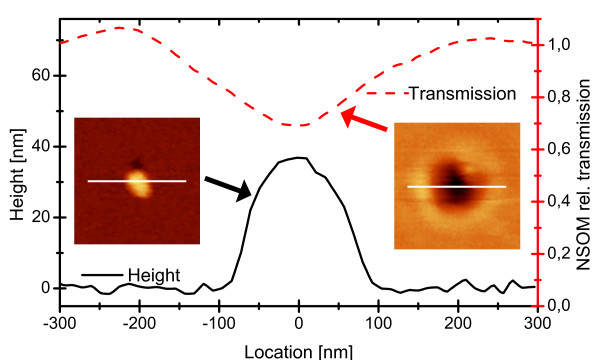
**Single Ag NP topography and relative transmission intensity.** This data was measured using a 458-nm excitation laser. Insets: topography / transmission image, white lines (600 nm length) mark the locations of the displayed curves.

Figure 1 shows the typical topography of an Ag NP embedded in aluminum oxide on the surface of a substrate (black solid line and left-hand inset). The corresponding NSOM intensity image (here using a wavelength of 458 nm) is also displayed in Figure 1 (right-hand inset). From these scans, we obtained a relative transmission^a^*T*_rel_(red dashed line in Figure 1) by normalizing the measured intensity *I*_m_ with the mean background intensity *I*_b_ from regions without particles: *T*_rel_=*I*_m_/*I*_b_. As shown in Figure 1*T*_rel_is decreased at the location of the NP; additionally, a halo of increased transmission is visible at approximately 200 nm distance from the central extinction peak. Similar effects have, in earlier studies, [8,9] been explained by interference of the light emitted from the NSOM tip and phase-shifted scattered light from the NP.

**Figure 2:**
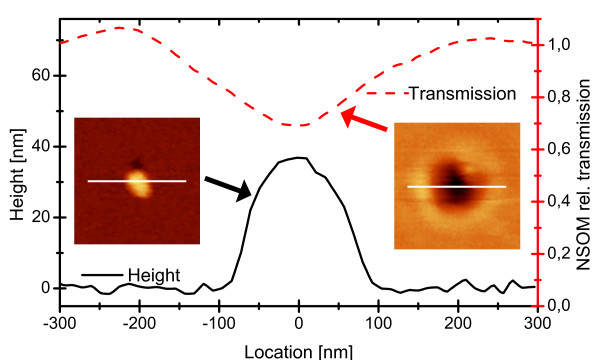
**Spectra for silver and polystyrene nanoparticles.** Upper part: NSOM extinction of spherical Ag NPs (black circles) and PS NPs (red triangles). Lower part: conventional extinction spectrum of spherical Ag NPs. Insets: details of realigning/search scans for 477 and 635 nm.

Scanning the same sample area with all available laser sources, spectrally resolved NSOM transmission can be obtained for single NPs. As the two insets in Figure 2 clearly show, smaller wavelengths (477 nm, left-hand side) are causing an intensity decrease at the position of the NP, while larger wavelengths (635 nm, right-hand side) may lead to an intensity increase. Converting for an individual NP the peak values of relative transmission (positive or negative) for each wavelength to a local change of extinction (relative optical density) by using *ΔOD*_NSOM_=−log_10_(*T*_rel_), the spectral behavior given as black solid circles in the upper panel of Figure 2 is obtained. For comparison, analogous measurements on PS NPs were processed in an identical way; the respective values are shown as red triangles in Figure 2.

## Results and discussion

As expected from previous works [10,11], the non-metallic PS particles lead to increased NSOM transmission (lower extinction) at any wavelength. While such a negative *ΔOD*_NSOM_ is also found for the Ag NPs at 635 and 785 nm, one observes in this case a contrast inversion towards smaller wavelengths with an extinction peak around 500 nm. This effect has been found to be due to the SPR of the Ag NPs [8,9,12], where the zero crossing (*ΔOD*_NSOM_=0) is located at the resonance. The position of contrast inversion cannot be extracted exactly from our experimental data but is apparently located at a wavelength of approximately 550 nm or larger. This means that the SPR obtained from the NSOM data is clearly deviating from the maximum extinction at approximately 475 nm wavelength measured in the conventional spectrum (shown in the lower panel of Figure 2). It is expected that the conductive probe tip causes a red shift of the SPR resonance compared to the far-field spectra due to near-field coupling effects [13], although we had a rather large distance of approximately 40 nm between the NPs and the tip due to the AlOx layer. The far-field peak position (lower panel of Figure 2) agrees well with our simulations with the finite element method [14] predicting an SPR at 470 nm for the refractive index (*n*≈1.66) of the ALD-generated AlOx layers. The band shape of the conventional spectrum is not clearly Lorentzian-like but exhibits some degree of inhomogeneous broadening, which might be caused by a distribution of NPs of different sizes or, possibly, some particle agglomerates also. In fact, a size distribution was visible in some of the topographic images. However, for this study, we restricted the NSOM scans to single NPs with a size of approximately 40 nm.

**Figure 3:**
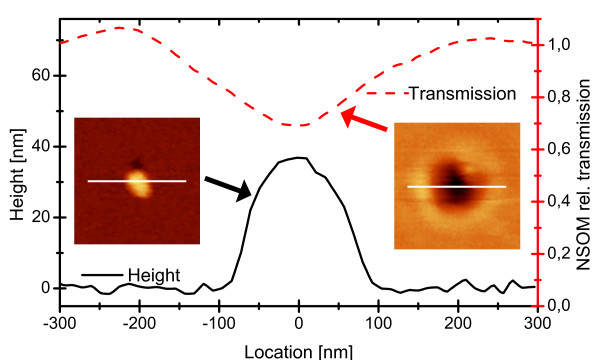
**Conventional difference spectrum.** Calculated from spectra measured with light polarized along the long and short axes of the reshaped Ag NPs; peaks are at 460 and 600 nm.

Analyzing the now femtosecond-laser-irradiated regions, one expects dichroism for NPs transformed to prolate spheroidal shape. Conventional spectra measured with different polarization (parallel and perpendicular to laser polarization, respectively: parallel to long or short NP axis) in irradiated regions only show very small differences which, however, are still considerably larger than the noise in the spectra. A corresponding difference spectrum (*ΔOD*=*OD*_long_−*OD*_short_) is presented in Figure 3. Clearly visible is a positive peak of *ΔOD*at about 600 nm, apparently due to the SPR of the long NP axis, and a negative peak at about 460 nm caused by the short-axis SPR. This gives evidence that the reshaping process has occurred. The isotropic background of the spectra, which has been removed by the subtraction of spectra for different polarization, indicates that only a fraction of the NPs have actually been transformed. For prolate spheroids, the observed peak positions are reproduced by our simulations, assuming an average aspect ratio of the NPs of 1.5 and a slightly (by approximately 5*%*) increased matrix refractive index. Such an index change is quite plausible considering that processes like Ag ion emission and strong local heating are believed to occur in the immediate NP surroundings upon femtosecond laser irradiation [6].

Trying to localize such an individual reshaped NP, we have conducted polarization-resolved NSOM scans. The optical path from the excitation laser to the NSOM cantilever is polarization maintaining, and the polarization of the incident light is maintained also in the near-field region of the probe tip [15]. Considering the above-discussed red shift in NSOM imaging, we can estimate the short-axis SPR to occur slightly below 550 nm and the long-axis SPR at 675 nm or above. Therefore, we chose the wavelength of 635 nm for pertinent NSOM scans. There, we are measuring at the long-wavelength side of the SPR using light polarized parallel to the short axis of the NP, whereas polarization along the long axis of the NP refers to the short-wavelength side, i.e., one expects a switch from local signal intensity increase to decrease upon 90° polarization rotation in the case of reshaped NPs.

**Figure 4:**
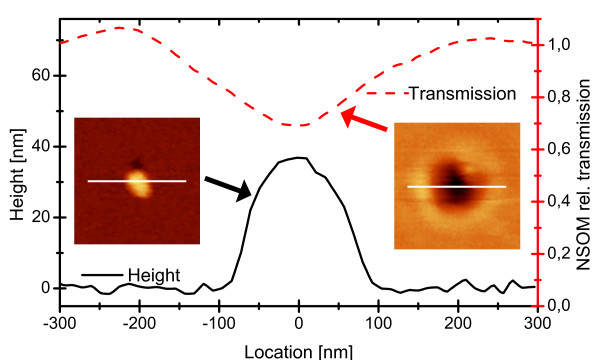
**Polarization-resolved NSOM scans.** Measured with the excitation laser polarized along the short/long axis of a femtosecond-irradiation-reshaped Ag NP. Insets: transmission images, each 750×750 nm^2^.

Figure 4 proves clearly that such a contrast inversion occurs: when the polarization of the 635 nm laser in the NSOM is parallel to the polarization of the femtosecond laser (and thus, parallel to the long NP axis), there is a significant decrease of transmission (red dashed curve and right-hand inset) at the location of the NP. In contrast, the signal recorded with polarization rotated by 90° shows a considerable transmission increase (black solid curve, left-hand image). Thus, a femtosecond-laser-reshaped Ag NP can be easily identified by observing contrast inversion upon polarization change at a suitable single excitation wavelength. By scanning larger sample areas successively with polarization parallel and perpendicular to the femtosecond laser polarization, we could verify the above-stated assumption that only a part of the Ag NPs have been transformed to prolate spheroids in our experiments. With our current laser sources, we were not able to determine by NSOM transmission measurements the actual SPR peak position for the long NP axis.

## Conclusions

In conclusion, we were able to show evidence for the spheroidal NP shapes after femtosecond irradiation by conventional spectroscopy and NSOM measurements. We further demonstrated the capabilities of polarization-resolved NSOM scans for detecting individual reshaped metallic nanoparticles, thus making it an excellent tool for a future in-depth analysis of the optical properties of single non-spherical NPs in dielectric matrices. In particular, we are planning to use femtosecond-laser-generated supercontinuum light for excitation in NSOM measurements to obtain spectrally resolved near-field images, promising to yield the complete spectral behavior of the NSOM signal around the plasmon resonance. On the theoretical side, numerical simulations of the near-field setup, including the metallic tip and its resonance shifting effects, are intended. The fact that only a small percentage of the particles are shape-transformed (also observed in other samples [7]) will also be a key issue of future investigations. The combination of femtosecond laser reshaping and polarization-resolved NSOM detection of single NPs also paves a possible route to a novel all-optical data storage technique with unprecedented storage density.

## Endnotes

^a^ This signal cannot be directly compared to classical far-field transmission since field enhancement on the NSOM tip and NP, as well as refractive index effects due to the bulge of the matrix around the NP, contribute to it.

## Competing interests

The authors declare that they have no competing interests.

## Authors’ contributions

MB prepared the samples, participated in the femtosecond laser irradiation, carried out the NSOM measurements, participated in the design of the study and drafted parts of the manuscript. CM carried out the theoretical simulations for the surface plasmon resonances and helped with the interpretation of the experimental data. HB performed in-depth ellipsometric analysis of AlOx layers created by ALD resulting in reliable optical index data and carried out the ALD processing. GS conceived of the study, participated in its design and drafted the manuscript. All authors read and approved the final manuscript.
